# Assessing functional impulsivity using functional near-infrared spectroscopy

**DOI:** 10.3389/fnrgo.2023.1207484

**Published:** 2023-12-01

**Authors:** Kenta Nakazawa, Kazue Hirabayashi, Wakana Kawai, Yasushi Kyutoku, Keith Kawabata Duncan, Ippeita Dan

**Affiliations:** ^1^Applied Cognitive Neuroscience Laboratory, Faculty of Science and Engineering, Chuo University, Tokyo, Japan; ^2^Shiseido Co., Ltd., MIRAI Technology Institute, Yokohama, Japan

**Keywords:** functional impulsivity, DLPFC, FPC, implicit experimental design, functional near infrared spectroscopy (fNIRS), neuromarketing, consumer neuroscience

## Abstract

**Introduction:**

In neuromarketing, a recently developing, inter-disciplinary field combining neuroscience and marketing, neurophysiological responses have been applied to understand consumers' behaviors. While many studies have focused on explicit attitudes, few have targeted implicit aspects. To explore the possibility of measuring implicit desire for a product, we focused on functional impulsivity related to obtaining a product as a reward and devised a product-rewarded traffic light task (PRTLT). The PRTLT requires participants to take risks under time pressure in order for them to maximize rewards in the form of commercial products, with the brand of products being an independent variable. Thus, we explored the feasibility of applying a PRTLT in a neuromarketing context to implicitly differentiate between the perceived value of products and supported our data with neurophysiological evidence obtained using fNIRS to concurrently monitor cortical activation.

**Methods:**

Thirty healthy students were asked to perform the PRTLT. We compared participants' functional impulsivity toward two different chocolate products that had obviously different values. Along with their behavioral responses, participants' cerebral hemodynamic responses during the PRTLT were measured using fNIRS covering the lateral prefrontal cortices and the neighboring regions. We conducted adaptive general linear model (GLM) analysis for hemodynamic responses. First, we identified the regions involved in the PRTLT. Second, we compared activation patterns between expensive and inexpensive conditions.

**Results:**

Behavioral analysis confirmed that the expensive condition trended toward producing a higher PRTLT score than did the inexpensive condition. fNIRS neuroimaging analysis showed task-derived activation in the bilateral dorsolateral prefrontal cortex (DLPFC) and frontopolar cortex (FPC). Moreover, we found significant differences between expensive and inexpensive conditions in the cortical activations in the FPC and the left-DLPFC.

**Conclusion:**

These results imply that the two products evoked different functional impulsivity, and the hemodynamic responses reflect that. Thus, we concluded that it is possible to observe differences in demand for products using a PRTLT that evokes functional impulsivity. The current study presents a new possibility in neuromarketing research of observing differences between consumers' covert attitudes toward commercially available products, possibly providing a neural basis related to hidden needs for some products.

## 1 Introduction

In recent years, neuromarketing, an inter-disciplinary field combining neuroscience and marketing, has been developing (Lee et al., 2007; Ariely and Berns, 2010; Morin, 2011; Plassmann et al., 2015; Meyerding and Mehlhose, 2020; Shahriari et al., 2020; Ma et al., 2021). This field attracts a great deal of interest because it may provide a deeper understanding, compared to traditional methods, of purchasing behavior by elucidating its underlying neural basis. The first clear neuromarketing study was reported in 2004; this fMRI study demonstrated that awareness of the brands of the beverages subjects consume might bias their preferences based on brand information and that the medial prefrontal cortex is the major neural substrate for such brand preference (McClure et al., 2004). While former psychological studies had presented similar results, which is to say that brand information dominates preference rating (Pronko and Bowles, 1948; Thumin, 1962; Woolfolk et al., 1983), the neuromarketing approach added neurophysiological evidence for the neural activity underlying common marketing approaches, such as questionnaires and behavioral experiments, cracking open the black box associated with cognitive processing. The neuromarketing approach, thereby, can elucidate the latent cognitive mechanisms of consumers' behaviors, such as feelings, emotions, values, memories, or judgments, through assessing neurophysiological responses without asking them directly (Lee et al., 2007; Ariely and Berns, 2010; Morin, 2011; Kopton and Kenning, 2014; Plassmann et al., 2015; Venkatraman et al., 2015; Ma et al., 2021).

While early studies suggested the potential of a neuromarketing approach, some studies have begun to exhibit its practical value. A previous study reported that prefrontal cortex activity was correlated with stimulus value when dieters engaged in real decisions about food consumption (Hare et al., 2009). Importantly, this cortical activity integrated both health and taste values in those who could exercise restraint in their choices, whereas it reflected only taste values in those who could not exercise restraint. Another study reported that dorsolateral prefrontal cortex (DLPFC) and orbitofrontal cortex (OFC) activity was related to the calculation of “Willingness To Pay (WTP) for goods” (Plassmann et al., 2007). It confirmed the relationship between WTP and brain regions and found a positive correlation between the medial orbitofrontal cortex and WTP. Additionally, the results of fMRI measurements were shown to provide a better predictor for advertising effectiveness than commonly used methods such as self-reports, implicit measures, eye tracking, biometrics, and electroencephalography (Venkatraman et al., 2015). Specifically, ventral striatum activation was the best predictor of responses to actual market advertising.

Owing to its detailed and high spatial resolution of whole-brain functional measurements, fMRI has been successfully applied in neuromarketing studies; however, a significant disadvantage exists. fMRI requires participants to be in a narrow, noisy scanner environment without moving in order to observe neural activation. However, measuring brain function with functional near-infrared spectroscopy (fNIRS), an optical method of monitoring hemodynamic responses of the lateral cerebral cortices, has great potential because it is not restricted by the serious limitations of fMRI. fNIRS is mobile, low cost, comfortable, and tolerant of body motion. It is also highly portable, and its importance has been noted and described as a major innovation in neuroeconomic research (Kopton and Kenning, 2014; Wilcox and Biondi, 2015; Meyerding and Mehlhose, 2020). In recent years, several studies have used fNIRS to concurrently examine brain activation during product evaluation using a WTP activity and reported that there was a positive intra-individual correlation between DLPFC activation and WTP scores (Kawabata Duncan et al., 2019; Hirabayashi et al., 2021). Another study reported that frontopolar cortex (FPC) activity was identified when making subjective value judgments during purchase decisions (Çakir Murat et al., 2018). This study also suggested that different mindsets related to budgets produce different activation patterns in the PFC. Another study reported that when a product was labeled, PFC activation increased significantly more than when the product was unlabeled during taste testing (Meyerding and Mehlhose, 2020). It showed the same pattern when taste tests were conducted with the same product actually being drunk but with different brand names shown. Particularly, there was a significant increase in PFC activation when the stronger brands were presented. These studies have demonstrated that fNIRS can provide neurophysiological evidence for the consumer's product evaluation and decision-making process.

In order to explore the neurophysiological bases of consumer-related behaviors, it is common for an experimental procedure to involve an explicit evaluation of a product during functional brain measurement. For example, the aforementioned WTP experiments required participants to explicitly evaluate their WTP for a target product. On the other hand, only a few experimental designs in neuroimaging-based neuromarketing research focus on an implicit cognitive component (e.g., Deppe et al., 2005). Various consumption behaviors are strongly influenced by factors outside of people's conscious awareness, and it is important to understand unconscious processing (Dimofte, 2010; Chartrand, 2011; Nosek et al., 2011). Notably, Friese et al. (2006) reported that actual behaviors and latent attitudes may differ, and emphasized that impulsive behavior and implicit measurement were important for the research because implicit preferences, rather spontaneous and uncontrolled behavior, may be particularly valuable for predicting purchasing behavior. This led us to focus on impulsivity, one important factor involved during decision making, and to assess it in an implicit experimental setting.

Impulsivity is classified into two types: functional and dysfunctional (Dickman, 1990). When impulsive behavior has the potential to work for the better, it is referred to as functional impulsivity. When it has detrimental results, it is classified as non-functional impulsivity. Burnett Heyes et al. (2012) developed a cognitive task called the “traffic light task” to assess functional impulsivity. This original traffic light task pushes participants to take risks under time pressure in order to maximize their reward. Briefly, participants first view a red light (stop signal), which successively turns yellow (preparation signal) with a variable duration, and finally turns green (go signal). Participants respond as rapidly as possible after the onset of the green light to obtain a reward because the value of the reward declines steeply; however, responding prior to the green light imposes a fixed penalty on the participant. Since the duration of yellow light varies across trials, participants cannot confidently predict the temporal onset of a green light.

The traffic light task was originally used to measure Individual differences in functional impulsivity. Burnett Heyes et al. (2012) revealed that better performance of the task correlated with a subscale in self-reported impulsivity. However, functional impulsivity might vary depending on the type of reward. Let us assume that a reward is given as a varying quantity of a product. If a participant highly valuates the product, we would expect the participant to have a higher degree of functional impulsivity and better performance on the traffic light task. In that case, if a participant is rewarded with a different product, the relative perceived value of the products might be assessed through differences in their performance of the traffic light task, allowing us to determine which product the participant implicitly prefers. Hence, we devised a product-rewarded traffic light task (PRTLT), in which a specific product image is presented during the yellow light period, and it is given as a reward depending on the participant's performance: better performance resulted in more of the product being rewarded. The PRTLT is expected to quantify functional impulsivity occurring upon presentation of a product as a reward, thereby allowing an estimation of perceived product value. As products, we selected chocolate because it proved to be highly preferred scoring above seven in p-point Likert scale in wide range of US population (Rozin et al., 1991). Also, in Japan, a recent commercial survey exhibited that 86% Japanese consumers like chocolate (Line Co., 2021). Based on its prevalent popularity, we expected chocolate products could evoke participant's desire to obtain it. Thus, using luxury and commodity chocolate products as the stimuli, we examined whether the PRTLT is applicable for marketing use.

Moreover, since the PRTLT has a high affinity for neuromarketing experimental settings using fNIRS, we also explored the neurophysiological basis of functional impulsivity by measuring cortical activation using fNIRS. However, it should be noted that although we may want to explore all the cognitive processes behind the PRTLT, fNIRS measurements are limited to certain parts of the lateral cerebral cortices. For the primary putative cognitive target, we focused on the yellow light period during which participants were expected to evaluate a presented product and decide how actively they will perform upon the onset of the green light. Since previous research has shown that prefrontal regions are activated when making evaluation and other decisions (Plassmann et al., 2007; Kawabata Duncan et al., 2019; Hirabayashi et al., 2021), we set fNIRS probes to cover the PFC. We first aimed to confirm that the cortical activation derived from the task would be observed. Then, after identifying the activated areas, we examined whether there were differences in the activation patterns between luxury and commodity product conditions. Combining our findings, we explored the feasibility of applying the PRTLT in a neuromarketing context to implicitly differentiate between the perceived value of products combined neurophysiological evidence obtained with concurrent fNIRS monitoring of cortical activation.

## 2 Method and materials

### 2.1 Participants

This experiment was conducted with thirty participants (all students, mean age 21.77 ± 1.25 years, 15 men and 15 women). We excluded one participant from the analyses since his evaluations of the products were outliers and significantly different than the evaluations of all other participants, resulting in 29 participants for the subsequent analyses. All participants were right-handed, and native speakers of Japanese. Before the experiment, informed consent was obtained from all participants. Participation was voluntary, and an honorarium as well as the chocolate rewards distributed according to their performance on the task were given to the participants after the experiment. The current study was in accordance with the latest version of the Helsinki Declaration. The current study was approved by the Institutional Ethics Committee of Chuo University.

### 2.2 Procedure

Participants were informed in advance that they should not eat or ingest caffeine for 2 h prior to the experiment. Before the fNIRS measurements, participants responded to two questionnaires. We conducted the Edinburgh's dominant hand test and confirmed that participants were right-handed (Oldfield, 1971). To recruit participants who have no serious medical or psychological problems, all participants were screened for major psychiatric disorders using the M.I.N.I. screen (Sheehan et al., 1998). We prepared two chocolate products from two brands; the expensive one pricing 500 yen per piece, and the inexpensive one pricing 20 yen per piece). We prepared three variations for each product resulting in six different chocolate product stimuli. These brands were chosen based on the assumption that the relationship between price and value for these brands is typically understood by consumers.

### 2.3 Experiment design

**Figure 1:**
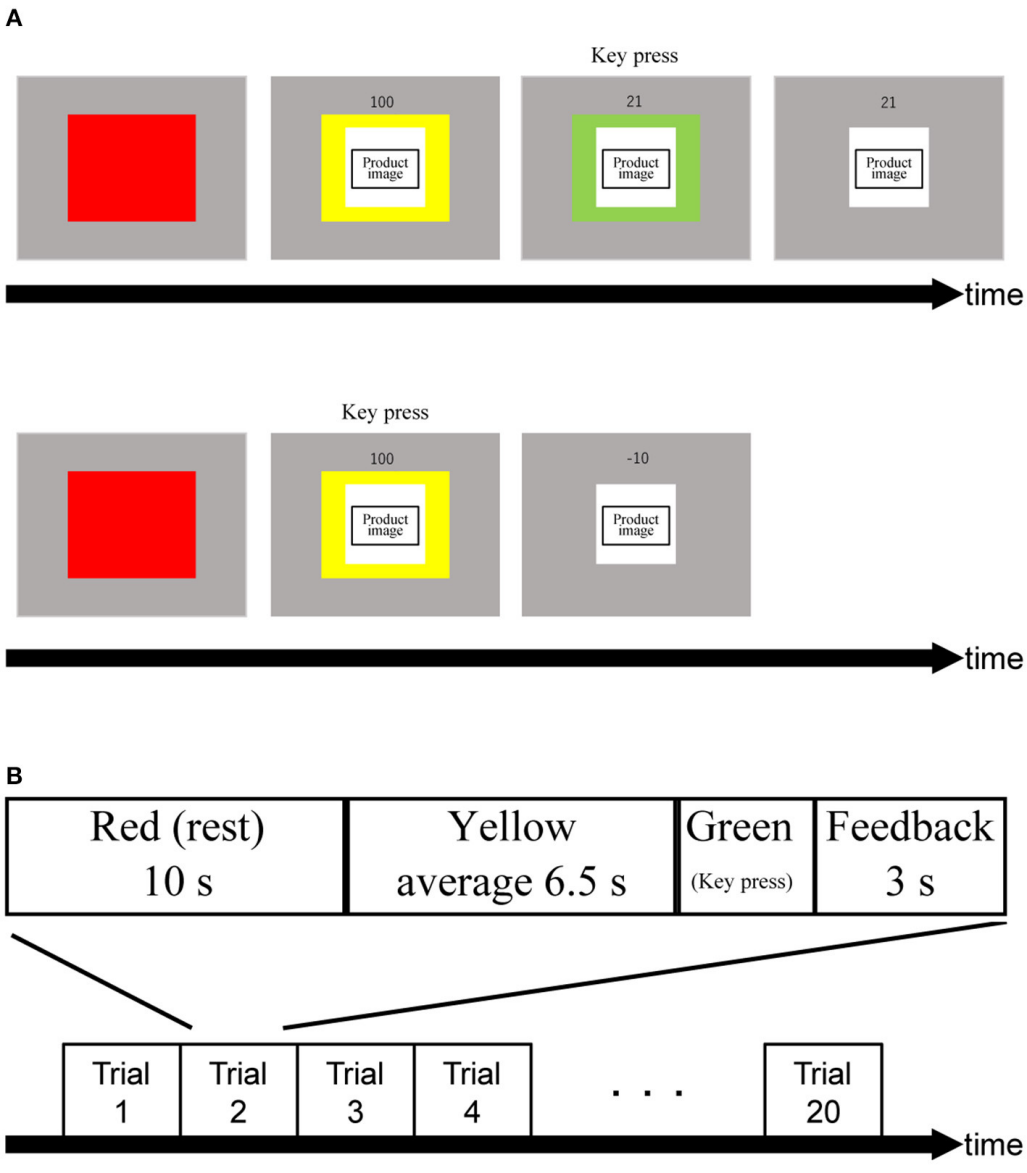
**(A)** Trial procedure. The order of product images was displayed randomly. Numbers above product images indicate relative rewards in percentage. Upon turning into green light, the relative reward indicator drops rapidly from 100% until a participant react to the green light. Consequently, obtained relative reward (in this example, 21) is shown. When a participant makes a false start, a small penalty is given (i.e.,−10). **(B)** Schematic showing an experimental block. The order of assembling and handling was random.

We designed the PRTLT (Figure 1) based on the Traffic Light Task (Burnett Heyes et al., 2012). Participants were asked to respond by pressing a key when the color of a cue presented with the image of a product changed from yellow to green, with an average interval of 6.5 s (SD: 0.25). For this task, there were two conditions (expensive/inexpensive). Participants performed both conditions 10 times each and in a random order. Subjects were informed in advance that their scores (S) would be based on their reaction times (RT in ms; Equation 1) but that they would be penalized for pressing the key before the color changed from yellow to green as in Burnett Heyes et al. (2012). To adjust for S to start from 100, we replaced the original numerator of 150 with 100. Thus, S starts from 100 at the onset of the green period, and rapidly decreases; for example, to the halfway point at 100 ms and the quarter point at 200 ms. Rewards were the same chocolates as we used in this test. S values were accumulative over the ten trials, adding points to the total score. Subjects were rewarded with one piece of chocolate per 50 points. We used the Psychophysics Toolbox in a MATLAB R2020a (Mathworks, Natick, MA) environment to create and operate these tasks (Brainard, 1997; Pelli, 1997; Kleiner et al., 2007).


(1) $$S=\frac{100}{e^{\frac{RT}{150}}}$$

### 2.4 fNIRS data acquisition

**Figure 2:**
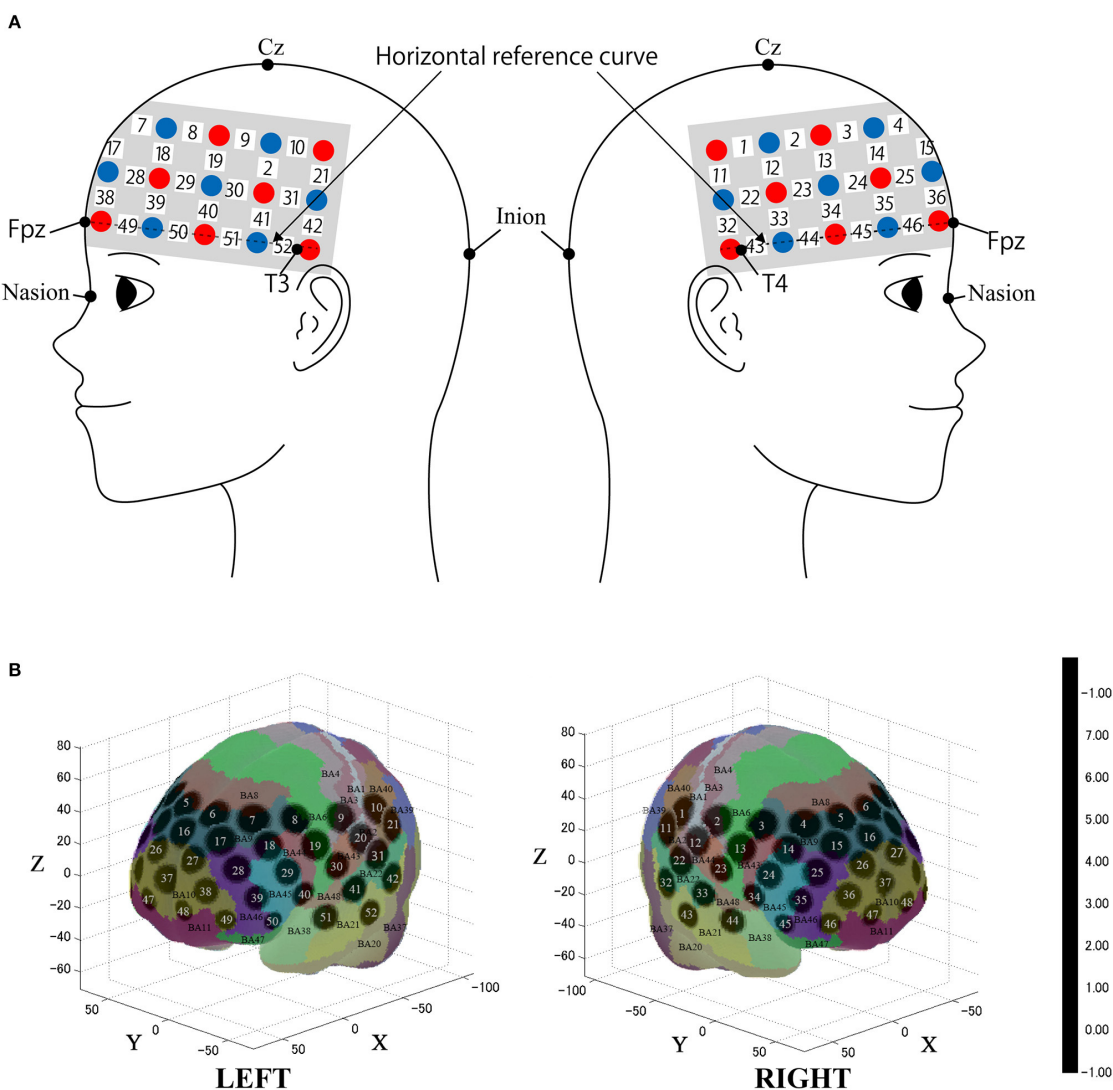
Spatial profiles of functional near infrared spectroscopy (fNIRS) channels. **(A)** The Figure shows left- and right-side views of the probe arrangement with fNIRS channel orientation. Detectors are indicated with blue circles, illuminators with red circles, and channels with white squares. **(B)** Corresponding channel numbers are shown in black. The estimated channel locations on the brain for both left- and right-side views are shown. The circles indicate the spatial variability associated with the estimation exhibited in Montreal Neurological Institute (MNI) space.

We used the multichannel continuous-wave fNIRS system ETG-4000 (Hitachi Medical Corporation, Kashiwa, Japan), making use of two wavelengths of near-infrared light (695 nm and 830 nm). We analyzed the fNIRS data using a modified Beer-Lambert law (Cope et al., 1988). This method enabled us to calculate signals reflecting the oxygenated hemoglobin (oxy-Hb), and deoxygenated hemoglobin (deoxy-Hb) concentration changes, obtained in units of millimolar × millimeter (mM × mm) (Maki et al., 1995). The sampling rate was set to 10 Hz. We placed the fNIRS probes to cover most of the prefrontal cortex together with dorsal parts of the temporal lobe and the anterior part of the parietal lobe as in previous studies (Oishi et al., 2023; Figure 2). We used 3 × 11 multichannel probe-holders that consisted of 17 illuminating and 16 detecting probes arranged alternately at an inter-probe distance of 3 cm for a total of 52 channels to obtain the cerebral hemodynamic responses. The probe was mounted according to the international 10–20 system as a reference point. First, the multichannel probe holder was placed such that the detector in the middle of the lowest row corresponded to Fpz. Then, the emitters and detectors in the lowest row were matched to the horizontal reference curve, which was determined by a straight line connecting T3-Fpz-T4 (Klem et al., 1999; Jurcak et al., 2007).

### 2.5 Registration of fNIRS channels to MNI space

After fNIRS measurements, the location of all the optodes and the landmarks, such as the nasion, inion, CZ, and bilateral preauricular reference points, were acquired using the Polhemus Patriot digitizer (Polhemus, Colchester, VT, USA). We employed probabilistic registration to register fNIRS data to MNI standard brain space (Tsuzuki et al., 2007; Tsuzuki and Dan, 2014). The spatial registration data were registered with macro-anatomical labeling (Okamoto et al., 2004; Okamoto and Dan, 2005) in reference to Brodmann's atlas (BA; Rorden and Brett, 2000).

### 2.6 Behavioral data analysis

To determine whether behavioral performance depended on each stimulus, we measured response time (RT). After the experiment, the total RT score was calculated for each condition (expensive and inexpensive), the calculated data were subjected to a paired *t*-test (two-tails) using IBM SPSS Statistics version 26 (IBM).

### 2.7 fNIRS data analysis

For the first level analysis, we used in-house codes on Matlab 2021a (The MathWorks, Inc., Natick, MA, USA) for fNIRS data analysis (available upon request). We used the oxy-Hb for analysis because the oxy-Hb signal is the most sensitive indicator of regional cerebral hemodynamic response (Huppert et al., 2006). First, each channel was pre-processed for oxy-Hb time series data. We preprocessed using temporal smoothing with convolution of the canonical hemodynamic response function (HRF) to the individual time series data (Friston et al., 2000). Channels with signal fluctuations of 10% or less were considered to have poor measurement quality and were excluded from the analysis. After exclusion, wavelet minimum description length (Wavelet-MDL) was applied to remove the effects of measurement noise, such as respiration and heart motion, from the remaining channels (Jang et al., 2009). After preprocessing, we conducted general linear model (GLM) analysis with regression of HRF on the oxy-Hb time series data from each channel for each subject. Basis functions used for GLM analysis were generated from the HRFs *h*(τ_p_, *t*) (Equation 2; Friston et al., 1998).


(2) $$h\left(\tau_{p},t\right)=\frac{t^{\tau_{p}}e^{-t}}{\left(\tau_{p}\right)!}-\frac{t^{\tau_{p}+\tau_{d}}e^{-t}}{A\left(\tau_{p}+\tau_{d}\right)!}$$

A previous study reported an adaptive GLM that yields the most effective HRF by selecting the optimal τ_p_ (first peak delay; Uga et al., 2014). Thus, in this study, we applied this method. We set τ_p_ from 6 s to 15 s to find an optimized τ_p_. We followed the default settings for the parameters, for example: τ_d_ (second peak delay): 10 s and A (amplitude ratio between the first and second peaks): 6. Basis functions *f* (τ_p_, *t*) (Equation 3) were generated by convolving the HRF *h*(τ_p_, t) with a boxcar function u(t).


(3) $$f\left(\tau_{p},t\right)=h\left(\tau_{p}\;,t\right)⊗u\left(t\right)$$

The ⊗ symbol denotes the convolution integral. The basis functions were used to compose each regressor. The first and second derivatives were included in order to further remove the influence of noise on individual data.

For predicting the hemodynamic response during a time series for each condition, we introduced four regressors. The first regressor consisted of a basis function covering the yellow, green, and feedback periods for each condition with its amplitude being presented as β_1_. The first and second derivatives of the basis function and a constant term are also used as regressors, and their β values were designated as β_2_, β_3_, and β_4_, respectively. We performed two rounds of regression analysis. The first analysis was to identify the cortical areas that were activated by the PRTLT in general. β values for this were designated as β_1-1st_, β_2-1st_, β_3-1st_, and β_4-1st_, respectively. Using the adaptive GLM, we searched for the optimal τ_p_ that maximized β_1-1st_ values: It was found that τ_p_ = 6 s. To see the brain activation tendencies for each task, the β_1-1st_ values for all channels were subjected to one-sample *t*-test against zero (two-tails). We chose channels in which significant differences were found as the region(s) of interest (ROI) to be used for the second analysis.

The second analysis was to see if different conditions led to different activation patterns in the areas where activation had been identified in the first analysis. β_1E-2nd_ β_1I-2nd_ are coefficients for the basis functions of the expensive and inexpensive conditions, respectively. β_2E-2nd_ and β_2I-2nd_ correspond to β values for the first derivatives. β_3E-2nd_ and β_3I-2nd_ correspond to β values for the second derivatives. β_4-2nd_corresponds to the constant terms. The τ_p_ for the second analysis was set to 6 s, which was same as for the first. To see whether the two conditions produced differences in the ROIs, the ROI β values for each condition were subjected to paired *t*-tests (two-tails).

Because we obtained 52 β values from the multi-channel fNIRS, we needed to consider family-wise errors (FWE) leading to a Type I error, which is when non-activating channels are incorrectly identified as significantly active. Since the first analysis examined whether or not each channel was activated during the PRTLT compared to rest conditions, we would expect relatively large activations. This could also produce a relatively high risk of Type I error. To reduce this risk, we adopted the Bonferroni method. For reference, we calculated a power under the conditions of sample size = 29 and one-sample *t*-test with α = 0.05/52, effect size = 0.8, and obtained a power of 0.72 using G^*^Power (release 3.1.9.7; Cohen, 1992; Faul et al., 2007). Therefore, we considered that the FWE correction could highly control Type I errors with small risk of Type II errors. As to the second analysis comparing the expensive and inexpensive conditions in activated areas, effect sizes were expected to be smaller than those in the first analysis, thereby Type I error risk should appropriately be controlled. Thus, we used the effective multiplicity (Meff) method utilizing eigenvalues derived from a correlation matrix of β values obtained from channels and subjects (Uga et al., 2015). Meff calculated for the second analysis was 5.23, and α = 0.0096 was adopted. When a medium effect size of 0.5 was assumed, it was expected to yield a power of 0.48 with a moderate risk of Type II error, whereas when large-medium effect size of 0.65 was assumed, the expected power became 0.76 with only a small risk of Type II errors.

## 3 Results

### 3.1 Behavioral analysis

**Figure 3:**
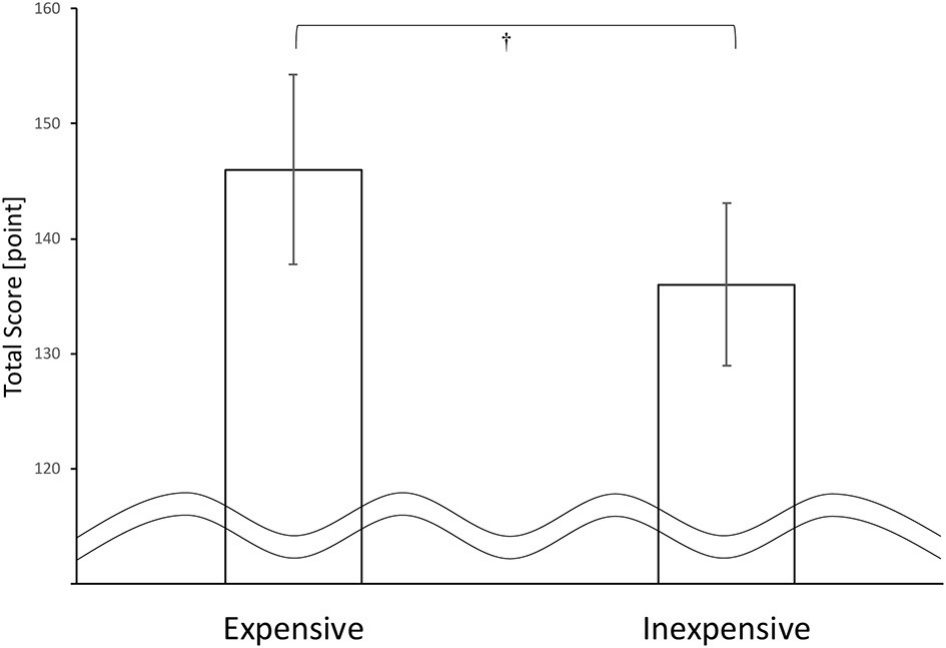
Total score for each condition. *N* = 29, error bars indicate *SE*, †indicates *p* < 0.1.

We performed a paired *t*-test (two-tails) on the PRTLT total scores between the expensive and inexpensive conditions. The results indicated a significant trend between conditions with a small-medium effect size as shown in Figure 3 (*t*_(28)_ = 1.98, *p* = 0.058, *d* = 0.37). Higher total score corresponded to faster reaction time (by 15 ms for each trial), and also to larger rewards, namely, 2.8 pieces of expensive chocolate compared to 2.3 pieces of inexpensive chocolate.

### 3.2 fNIRS analysis

**Figure 4:**
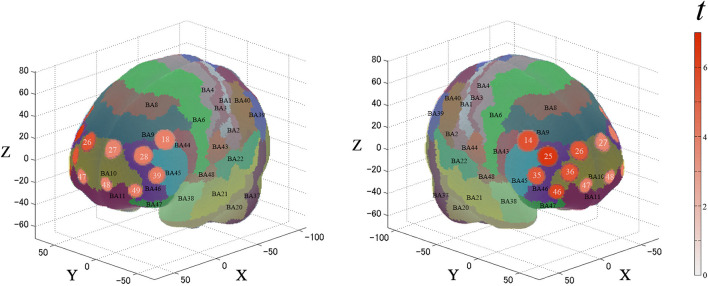
Results of the cortical activation patterns for the PRTLT task. Activated regions are shown according to the color bar.

For the first analysis, we performed a one-sample *t*-test against zero (two-tails) on the β-values for all channels to examine cortical activation patterns during this task. Figure 4 and Table 1 describe the results. During the task, the significantly activated channels were as follows: channels 25 and 35 (right-DLPFC); channels 28 and 39 (left-DLPFC); channels 26, 27, 36, 46, 47, 48, and 49 (FPC); channel 14 (right Broca's area); and channel 18 (left Broca's area).

**Table 1:** Estimated most likely locations of PRTLT-activated channels based on the probabilistic registration method.

	**MNI coordinates**			**Macro-anatomy**			**Activation**		
**CH**	**x**	**y**	**z**	***SD***	**Brodmann Area**	**%**	***t***	***p***	***d***
14	47.7	34.7	38.3	10.6	R-(mirror) Broca's area (BA45)	42.3	5.65	< 0.001	1.07
18	43.7	34.7	38.7	10.3	L-Broca's area (BA45)	37.1	4.06	< 0.001	0.77
25	41.3	52.3	27.3	10.0	R-DLPFC (BA46)	80.4	6.57	< 0.001	1.24
26	16.7	65.7	29.3	10.1	FPC (BA10)	82.5	5.50	< 0.001	1.04
27	11.7	65.0	29.7	10.0	FPC (BA10)	80.4	3.97	< 0.001	0.75
28	36.7	53.7	27.7	9.7	L-DLPFC (BA46)	96.8	4.27	< 0.001	0.80
35	51.0	48.3	11.3	8.9	R-DLPFC (BA46)	64.1	5.40	< 0.001	1.02
36	30.3	67.3	13.7	9.2	FPC (BA10)	97.9	5.51	< 0.001	1.04
39	48.0	48.7	11.3	8.6	L-DLPFC (BA46)	63.5	4.28	< 0.001	0.81
46	41.3	62.7	−1.7	7.9	FPC (BA10)	76.0	6.14	< 0.001	1.16
47	16.7	73.0	0.3	8.2	FPC (BA10)	68.3	4.24	< 0.001	0.80
48	12.3	74.0	0.7	7.7	FPC (BA10)	74.0	3.98	< 0.001	0.75
49	37.3	63.7	−1.3	7.2	FPC (BA10)	91.4	4.19	< 0.001	0.79

Values for x, y, and z correspond to the most likely coordinate values in MNI standard coordinate systems. SD is standard deviation associated with spatial registration in MNI coordinate values. Macro-anatomical estimations based on the Brodmann Atlas are indicated with accuracy probability (%). Cortical activations observed via oxy-Hb hemodynamic response are shown with the following values: t = t-values; p = p-values; d = Cohen's d.

**Figure 5:**
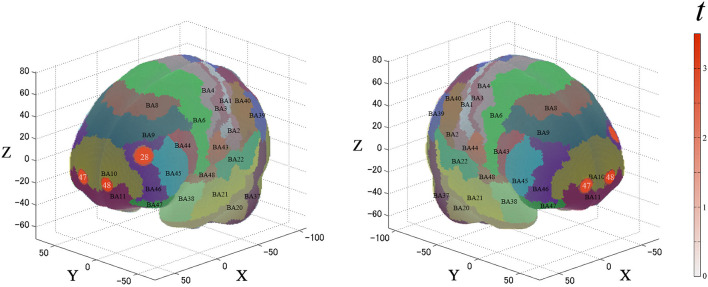
Results of the cortical activation contrast between conditions (expensive–inexpensive). Activated regions are shown according to the color bar.

For the second analysis, we performed a paired *t*-test (two-tails) on the β-values for the ROIs. Figure 5 and Table 2 describe the results. Between each condition, the significantly activated channels were as follows: channel 28 (left-DLPFC) and channels 47 and 48 (FPC).

**Table 2:** Estimated most likely locations of PRTLT-activated channels based on the probabilistic registration method for the contrast between expensive and inexpensive conditions.

	**MNI coordinates**			**Macro-anatomy**			**Activation**		
**CH**	**x**	**Y**	**z**	***SD***	**Brodmann Area**	**%**	***t***	***p***	***d***
28	−36.7	53.7	27.7	9.7	L-DLPFC (BA46)	96.8	2.87	0.008	0.54
47	16.7	73.0	0.3	8.2	FPC (BA10)	68.3	2.96	0.006	0.56
48	−12.3	74.0	0.7	7.7	FPC (BA10)	74.0	3.12	0.004	0.59

Values for x, y, and z correspond to the most likely coordinate values in MNI standard coordinate systems. SD is standard deviation associated with spatial registration in MNI coordinate values. Macro-anatomical estimations based on the Brodmann Atlas are indicated with accuracy probability (%). Cortical activations observed via oxy-Hb hemodynamic response are shown with the following values: t = t-values; p = p-values; d = Cohen's d.

For reference purposes, a detailed summary of the data from all channels that measured both hemoglobin species (oxy- and deoxy-Hb) are presented in the Supplementary material.

## 4 Discussion

### 4.1 Summary of the results

The purpose of this study was to clarify whether it is possible to measure implicit desire for a product using the PRTLT, a cognitive task involving functional impulsivity. Behavioral analysis confirmed that the expensive condition trended toward higher scores than did the inexpensive condition. Functional neuroimaging analysis revealed bilateral DLPFC and FPC activation as task-derived activation. In addition, a comparison between conditions using these as ROIs confirmed the activation of the left-DLPFC and FPC for the expensive condition.

### 4.2 Interpretation of behavioral data

In the current experiment, there was a trend toward significance in the PRTLT score, which depended on reaction time, between conditions. The effect size was small-medium. These behavioral results implies that PRTLT may be used as a behavioral measure in the current form when small-medium effect size is allowed. However, to increase the statistical power, a larger sample size or modification of experimental design is necessary. One possible concern was the selection of chocolate as stimuli. As indicated by Rozin et al. (1991), chocolate is highly preferred diet, and even inexpensive one was well desired and evoked moderate functional impulsivity. Selection of highly desired and lowly desired products might have more marked difference in behavioral measurement. Based on this, we will proceed with the discussion on fNIRS data with the assumption that functional impulsivity was moderately enhanced by the desire for the product.

### 4.3 Interpretation of fNIRS data

As expected, we observed bilateral prefrontal activations during the PRTLT task in general. Furthermore, concerning the contrast between the expensive and inexpensive conditions, the left DLPFC and bilateral FPC were significantly more activated in the expensive condition. Since this is the first study to apply fNIRS neuroimaging to the PRTLT, we have refrained from presenting strong hypotheses about cortical activation patterns. Considering the highly exploratory nature of the current study, we have attempted to interpret the current results based on several cognitive processes involved in the PRTLT. Here we will start discussing fNIRS data based on the cognitive demands related to the PRTLT. The present task is considered to require the following cognitive elements: evaluation of the product at the time of “yellow” presentation, motivation to and inhibition against pressing the button, judgment of the color (red, yellow, green), and feedback on the sequence of actions. Based on these cognitive elements, we will consider a functional interpretation of brain activation during the PRTLT.

First, bilateral DLPFC activation was observed. Previous studies have shown activation of the bilateral DLPFC during decision making (Rao et al., 2008; Cazzell et al., 2012; Schommartz et al., 2021). Another study investigated risk-related decision making using tDCS, which increases DLPFC activity, and found that bilateral DLPFC was associated with an aversion to risk-taking response (Fecteau et al., 2007). Moreover, studies have reported that the right-DLPFC was activated when making an WTP evaluation (Plassmann et al., 2007; Kawabata Duncan et al., 2019; Hirabayashi et al., 2021). Therefore, the activation of the right-DLPFC observed in the current study is considered relevant for the PRTLT as it is a cognitive task involving the valuation of presented products to control risk-taking behavior.

Regarding the left-DLPFC, there have been many reports showing activation related to decision-making, including value forecasting (Heekeren et al., 2006; Hare et al., 2009; Kahnt et al., 2011; Harlé and Sanfey, 2012). In particular, the left-DLPFC was shown to be recruited when self-control is required during overall evaluation (Hare et al., 2009). In executing the PRTLT, the participants were likely to implicitly evaluate the presented products, and this was reflected in marginal difference in RT for two chocolate brands. In addition, since the PRTLT by nature involves behavioral inhibition of responses during the task, we considered that the left-DLPFC activation observed was relevant to the PRTLT in general. That there was greater left-DLPFC activation in the expensive condition is also in line with this notion because there might have been a higher demand for self-control for the more expensive product.

With respect to the activation of the FPC, it has been recognized that the FPC plays a role in switching attention (Pollmann, 2001; Boorman et al., 2009; Laureiro-Martínez et al., 2015). A previous study reported that the FPC is related to calculating subjective values and making purchasing behavior decisions (Çakir Murat et al., 2018). Another study showed that brain stimulation targeting the FPC increased the amount of effort participants were willing to exert for rewards, implying that it is involved in maintaining motivation to achieve goals (Soutschek et al., 2018). As a task in general, the PRTLT involves mode switching from yellow to green signal states, subjective valuation of a product, and maintaining motivation to obtain large rewards. Thus, in view of the above listed studies on FPC functions, it is considered relevant that the FPC was recruited for the PRTLT. It is also reasonable that the FPC activation was enhanced in the expensive condition because there would have been higher demand for the product entailing greater attention to task switching and higher motivation for obtaining the reward.

As discussed above, the cortical activation patterns for the PRTLT in general and those for the contrast in price difference of the reward were mostly relevant considering the specific cognitive processes involved in the PRTLT under different conditions. However, we must be careful to avoid in-depth discussions about these results in order to avoid reverse inference (Poldrack, 2006). For further validation of the interpretation of the cortical activations involved in the PRTLT, detailed experiments focusing on specific cognitive components of the PRTLT are necessary.

### 4.4 Limitations and future perspectives

This study utilized, as stimuli, branded products with widely varying prices to make a clear difference in demand. The PRTLT succeeded in revealing an implicit differentiation between expensive and inexpensive products marginally through behavioral measurements and significantly through cortical activation patterns in the FPC and the left-DLPFC. As demonstrated in this study, the PRTLT has substantial potential as a neuromarketing method to implicitly assess consumers' functional impulsivity toward obtaining a product depending on their evaluation of the product as a reward. However, we have to note that there are several issues to be resolved regarding the practical application of the PRTLT. First, to test the practical utility of the task, we selected two products with obviously different commercial values, which can easily be used as rewards. However, it may not always be the case that we wish to test two or more products with clear differences in value. We have to examine whether the PRTLT is applicable for evaluating differences in functional impulsivity between products with similar values. Second, as the task name implies, the materialistic product itself was the reward. Thus, the current experimental conditions can only allow the evaluation of products within a budgetary limit. Meanwhile, evaluation of genuinely expensive products, such as luxury bags and automobiles, and consumer services, such as restaurant use and hotel stays, may not be feasible with the current experimental design. One plausible option may be to utilize indirect monetary rewards, but this would need further verification. Third, the effect size of the PRTLT may not be large enough for actual neuromarketing application. The behavioral scores yielded a small-medium effect size and cortical activation yielded a medium effect size. With the given effect sizes, several dozen consumers would be required to assess products with marked value differences. Despite these limitations, the current experimental design is applicable for the neuromarketing evaluation of commodity products as long as they can be given as rewards to the participants.

## 5 Conclusion

Our aim was to differentiate products' implicit values using a task that induces functional impulsivity. The results revealed that the two products yielded marginal differences in behavioral scores, reflecting in RT, and significant differences in cortical activations in the FPC and the left-DLPFC. This implies that the two products evoked different functional impulsivity, and the hemodynamic responses reflect that. Thus, we conclude that it is possible to observe differences in desire for products using the PRTLT to evoke functional impulsivity. Most designs currently used in neuromarketing show differences in consumers' overt attitudes to products. Compared to existing methods, the PRTLT we propose here could evaluate potential demands of consumers based on implicit measurement with a solid neural basis underlying behavioral assessment. Taken together, we propose a new possibility in neuromarketing research for showing differences in consumers' covert attitudes toward commercially available products, possibly providing a neural basis related to hidden needs for some products.

## 6 Significance to *neuroergonomics*

In recent years, neuromarketing, an inter-disciplinary field combining neuroscience and marketing, has been developing. While neurophysiological responses have been applied in an attempt to understand consumer behavior, many research studies have focused on explicit attitudes, and few studies have targeted implicit aspects. To explore the possibility of measuring implicit demand for a product, we focused on functional impulsivity for obtaining a product as a reward and devised a product-rewarded traffic light task (PRTLT), which is an adaptation of the original traffic light task (TLT) to measure the functional impulsivity of a person. The TLT requires participants to take risks under time pressure in order to maximize their reward. In the PRTLT, we made the reward a variable and compared participants' functional impulsivity toward two different chocolate products with obviously different values. Thus, we explore the feasibility of applying the PRTLT in a neuromarketing context to implicitly differentiate between the perceived value of products, with behavioral response supported by neurophysiological evidence obtained through concurrent fNIRS monitoring of cortical activation. Behavioral analysis confirmed that the expensive condition trended toward higher scores than did the inexpensive condition. fNIRS neuroimaging analysis revealed bilateral DLPFC and FPC activation as task-derived activation. In addition, we found significant differences in cortical activations in the FPC and the left-DLPFC. This implies that the two products evoked different functional impulsivity, and the hemodynamic responses reflect that. It also implies that it is possible to observe differences in demands for products using the PRTLT to evoke functional impulsivity. While most designs currently used in neuromarketing show differences in consumers' overt attitudes toward products, the PRTLT we propose here can evaluate potential demands of consumers based on implicit measurements with a solid neural basis underlying behavioral assessment. Thus, we propose a new possibility for neuromarketing research: observing differences between consumers' covert attitudes toward commercially available products while possibly providing a neural basis related to hidden needs for some products.

## Data availability statement

The raw data supporting the conclusions of this article will be made available by the authors, without undue reservation.

## Ethics statement

The studies involving humans were approved by Institutional Ethics Committee of Chuo University. The studies were conducted in accordance with the local legislation and institutional requirements. The participants provided their written informed consent to participate in this study.

## Author contributions

KK, KH, and KN contributed to the conception and design of the study. KN and KH performed the experiments, analyzed data, and performed statistical analyses. YK and KN developed the statistical analysis plan. KN and ID wrote the manuscript. WK performed data analyses and prepared supplementary material. KK and ID supervised this study. All authors have reviewed the manuscript and approved the final version for publication.
