# Supplementary material for: Assessing functional impulsivity using functional near-infrared spectroscopy
Source: Front Neuroergon. 2023 Dec 1;4:1207484. doi: 10.3389/fnrgo.2023.1207484 (PMC10790886; doi:10.3389/fnrgo.2023.1207484)
Supplement: Supplementary file 1 [file Data_Sheet_1.pdf]

Supplementary Materials

Assessing Functional Impulsivity Using Functional Near-Infrared Spectroscopy

Kenta Nakazawa[1], Kazue Hirabayashi[2], Wakana Kawai[1], Yasushi Kyutoku[1], Keith Kawabata Duncan[2], Ippeita Dan[1*]

*** Correspondence:** Corresponding Author: dan@brain-lab.jp

1 Supplementary Tables

Supplementary Table 1 Estimated most likely locations of all channels using the probabilistic registration method.

CH	MNI-coordinates				Anatomy	Prob.
	x	y	z	SD		%
1	65.0	-36.3	48.3	11.5	R-Wernicke's area (BA40)	82%
2	60.7	-10.3	48.3	11.5	R-M1 (BA4)	32%
3	50.0	15.7	49.7	11.3	R-DLPFC (BA9)	60%
4	33.7	34.7	50.7	11.6	R-DLPFC (BA9)	75%
5	13.7	45.7	53.0	11.4	R-DLPFC (BA9)	69%
6	-9.7	45.7	52.7	11.5	L-DLPFC (BA9)	65%
7	-30.3	33.3	51.3	11.7	L-DLPFC (BA9)	67%
8	-47.0	15.3	51.3	10.9	L-DLPFC (BA9)	62%
9	-58.3	-10.7	48.7	10.8	L-M1 (BA4)	34%
10	-62.3	-36.7	48.7	11.3	L-Wernicke's area (BA40)	81%
11	66.0	-46.7	35.7	12.3	R-Wernicke's area (BA40)	69%
12	69.0	-18.7	35.7	10.5	R-S1 (BA2)	49%
13	61.0	9.3	36.3	10.8	R-Pre-SMA (BA6)	63%
14	47.7	34.7	38.3	10.6	R-Broca's area (BA45)	42%
15	26.3	52.3	40.3	11.1	R-DLPFC (BA9)	79%
16	3.3	56.7	40.3	11.1	R-DLPFC (BA9)	85%
17	-22.0	52.3	41.0	11.0	L-DLPFC (BA9)	84%
18	-43.7	34.7	38.7	10.3	L-Broca's area (BA45)	37%
19	-57.7	9.7	36.7	10.6	L-Pre-SMA (BA6)	59%
20	-66.7	-19.3	35.7	10.4	L-S1 (BA2)	52%

21	-65.0	-46.7	36.7	11.6	L-Wernicke's area (BA40)	75%
22	71.0	-30.7	21.3	11.1	R-STG (BA22)	51%
23	68.0	0.3	23.3	9.1	R-Subcentral area (BA43)	71%
24	57.7	29.7	24.3	9.5	R-Broca's area (BA45)	92%
25	41.3	52.3	27.3	10.0	R-DLPFC (BA46)	80%
26	16.7	65.7	29.3	10.1	PFC (BA10)	83%
27	-11.7	65.0	29.7	10.0	PFC (BA10)	80%
28	-36.7	53.7	27.7	9.7	L-DLPFC (BA46)	97%
29	-54.3	29.7	24.3	9.4	L-Broca's area (BA45)	93%
30	-65.3	-0.3	23.3	9.4	L-Subcentral area (BA43)	73%
31	-69.0	-30.7	23.3	11.1	L-STG (BA22)	37%
32	72.0	-41.7	4.7	10.8	R-STG (BA22)	58%
33	71.0	-11.7	5.3	9.1	R-STG (BA22)	86%
34	62.0	21.3	10.3	8.9	R-Broca's area (BA45)	43%
35	51.0	48.3	11.3	8.9	R-DLPFC (BA46)	64%
36	30.3	67.3	13.7	9.2	PFC (BA10)	98%
37	4.0	70.0	15.0	10.0	PFC (BA10)	100%
38	-25.7	67.7	14.7	8.8	PFC (BA10)	99%
39	-48.0	48.7	11.3	8.6	L-DLPFC (BA46)	64%
40	-59.0	21.3	9.7	8.9	L-Broca's area (BA45)	42%
41	-67.3	-12.3	6.7	9.9	L-STG (BA22)	80%
42	-70.0	-41.7	6.3	10.8	L-STG (BA22)	63%
43	73.0	-22.7	-10.3	9.1	R-MTG (BA21)	99%
44	64.3	5.7	-8.7	9.1	R-MTG (BA21)	54%
45	56.0	40.3	-3.7	7.2	R-Broca's area (BA45)	56%
46	41.3	62.7	-1.7	7.9	FPC (BA10)	76%
47	16.7	73.0	0.3	8.2	FPC (BA10)	68%
48	-12.3	74.0	0.7	7.7	FPC (BA10)	74%
49	-37.3	63.7	-1.3	7.2	FPC (BA10)	91%
50	-54.0	40.7	-3.7	6.7	L-Broca's area (BA45)	59%
51	-62.0	5.7	-8.3	9.4	L-MTG (BA21)	48%
52	-71.0	-22.3	-10.3	9.2	L-MTG (BA21)	96%

Supplementary Table 2 Oxy-Hb hemodynamic responses assessed using one-sample t-tests on the PRTLT task for all channels.

CH	mean	*SD*	*t*	*P*	*d*
1	0.014	0.027	2.80	0.009	0.529
2	0.019	0.045	2.26	0.032	0.427
3	0.008	0.032	1.42	0.168	0.268
4	0.016	0.037	2.34	0.027	0.442
5	0.009	0.031	1.65	0.110	0.312
6	0.009	0.020	2.47	0.020	0.467
7	0.008	0.027	1.68	0.104	0.318
8	0.012	0.030	2.16	0.040	0.408
9	0.012	0.037	1.72	0.097	0.325
10	0.011	0.029	2.09	0.046	0.394
11	0.020	0.034	3.17	0.004	0.598
12	0.009	0.063	0.81	0.428	0.152
13	0.017	0.040	2.30	0.029	0.434
14	0.029	0.028	5.65	**<.001**	1.068
15	0.025	0.039	3.47	0.002	0.655
16	0.019	0.034	2.93	0.007	0.553
17	0.014	0.022	3.40	0.002	0.642
18	0.025	0.033	4.06	**<.001**	0.768
19	0.004	0.032	0.76	0.453	0.144
20	-0.005	0.047	-0.61	0.549	-0.115
21	0.015	0.033	2.55	0.017	0.481
22	0.012	0.051	1.26	0.217	0.239
23	0.012	0.058	1.16	0.258	0.218
24	0.027	0.045	3.20	0.003	0.604
25	0.041	0.034	6.57	**<.001**	1.241
26	0.031	0.031	5.50	**<.001**	1.040
27	0.030	0.040	3.97	**<.001**	0.750
28	0.031	0.039	4.24	**<.001**	0.801
29	0.019	0.033	3.03	.005	0.572
30	-0.007	0.064	-0.57	.572	-0.108
31	0.004	0.044	0.45	.659	0.084
32	0.016	0.048	1.78	.085	0.337
33	0.012	0.062	1.02	.315	0.193

34	0.023	0.057	2.18	.038	0.413
35	0.033	0.033	5.40	**<.001**	1.020
36	0.039	0.038	5.51	**<.001**	1.042
37	0.032	0.049	3.47	.002	0.657
38	0.032	0.056	3.07	.005	0.580
39	0.031	0.039	4.28	**<.001**	0.809
40	0.001	0.055	0.08	.936	0.015
41	0.004	0.066	0.30	.766	0.057
42	-0.005	0.049	-0.56	.583	-0.105
43	0.010	0.059	0.87	.393	0.164
44	0.016	0.069	1.28	.210	0.242
45	0.027	0.049	3.02	.005	0.570
46	0.040	0.035	6.14	**<.001**	1.161
47	0.037	0.047	4.24	**<.001**	0.801
48	0.039	0.053	3.98	**<.001**	0.751
49	0.037	0.048	4.19	**<.001**	0.792
50	0.027	0.048	2.97	.006	0.561
51	0.014	0.081	0.90	.377	0.170
52	0.006	0.078	0.40	.692	0.076

Notes. p values of significant channels ($p < \alpha$ (= .05) / 52) are in boldface. *SD* = standard deviation of spatial estimates; *t* = *t*-values; *p* = *p*-values; *d* = Cohen's *d*.

Supplementary Table 3 Deoxy-Hb hemodynamic responses assessed using one-sample t-tests on the PRTLT task.

CH	mean	*SD*	*t*	*P*	*d*
1	-0.001	0.011	-0.49	.631	-0.09
2	0.000	0.027	-0.10	.922	-0.02
3	-0.010	0.021	-2.51	.018	-0.47
4	-0.001	0.010	-0.80	.432	-0.15
5	0.000	0.009	-0.10	.918	-0.02
6	-0.003	0.011	-1.26	.219	-0.24
7	0.000	0.010	0.10	.922	0.02
8	0.003	0.010	1.69	.103	0.32
9	0.001	0.013	0.38	.709	0.07
10	-0.006	0.010	-3.03	.005	-0.57
11	-0.004	0.013	-1.75	.091	-0.33
12	0.000	0.018	-0.03	.976	-0.01
13	-0.008	0.021	-1.99	.057	-0.38
14	0.002	0.011	1.14	.265	0.21
15	0.000	0.009	-0.06	.955	-0.01
16	0.003	0.018	0.83	.416	0.16
17	0.004	0.015	1.57	.128	0.30
18	0.000	0.009	-0.25	.802	-0.05
19	0.003	0.012	1.47	.153	0.28
20	0.004	0.022	0.98	.336	0.19
21	-0.003	0.013	-1.28	.212	-0.24
22	0.001	0.023	0.14	.892	0.03
23	0.002	0.025	0.37	.712	0.07
24	-0.001	0.012	-0.24	.813	-0.05
25	0.000	0.010	-0.15	.885	-0.03
26	0.003	0.011	1.45	.159	0.27
27	0.007	0.010	3.76	**.001**	0.71
28	0.001	0.013	0.49	.631	0.09
29	0.003	0.008	1.90	.068	0.36
30	0.002	0.033	0.31	.756	0.06
31	-0.001	0.028	-0.11	.917	-0.02
32	-0.013	0.027	-2.55	.017	-0.48
33	-0.003	0.026	-0.66	.518	-0.12

34	-0.005	0.021	-1.15	.258	-0.22
35	0.003	0.011	1.35	.187	0.26
36	0.006	0.013	2.58	.015	0.49
37	0.011	0.020	2.96	.006	0.56
38	0.010	0.016	3.34	.002	0.63
39	0.005	0.008	3.09	.004	0.58
40	0.004	0.028	0.84	.408	0.16
41	0.000	0.046	0.05	.961	0.01
42	-0.001	0.028	-0.21	.837	-0.04
43	-0.003	0.033	-0.41	.683	-0.08
44	-0.003	0.030	-0.57	.574	-0.11
45	0.001	0.021	0.35	.726	0.07
46	0.005	0.012	2.52	.018	0.48
47	0.010	0.013	4.42	**<.001**	0.84
48	0.013	0.014	4.72	**<.001**	0.89
49	0.008	0.016	2.68	.012	0.51
50	0.009	0.018	2.54	.017	0.48
51	0.001	0.038	0.13	.897	0.02
52	0.003	0.033	0.48	.634	0.09

Notes. p values of significant channels ($p < \alpha$ (= .05) / 52) are in boldface. *SD* = standard deviation of spatial estimates; *t* = *t*-values; *p* = *p*-values; *d* = Cohen's *d*.

Supplementary Table 4 Oxy-Hb hemodynamic response contrast between expensive and inexpensive conditions assessed using paired t-tests for channels activated by PRTLT.

CH	mean diff.	SD	t	p	d
14	0.007	0.045	0.84	.408	0.16
18	0.014	0.037	1.95	.062	0.37
25	0.018	0.045	2.16	.040	0.41
26	0.017	0.041	2.18	.038	0.41
27	0.022	0.046	2.63	.014	0.50
28	0.020	0.038	2.87	**.008**	0.54
35	0.006	0.045	0.70	.489	0.13
36	0.014	0.038	1.96	.060	0.37
39	0.013	0.040	1.71	.098	0.32
46	0.011	0.036	1.64	.112	0.31
47	0.019	0.035	2.96	**.006**	0.56
48	0.028	0.048	3.12	**.004**	0.59
49	0.019	0.047	2.16	.040	0.41

Notes. p values of significant channels ($p < \alpha$ (= .05) / Meff value) are in boldface. Meff value = 5.23. *SD* = standard deviation of spatial estimates; *t* = *t*-values; *p* = *p*-values; *d* = Cohen's *d*.

Supplementary Table 5 Deoxy-Hb hemodynamic response contrast between expensive and inexpensive conditions assessed using paired t-tests for channels presented in Supplementary Table 4.

CH	mean diff.	SD	t	p	d
14	-0.005	0.013	-1.99	.056	-0.38
18	-0.004	0.010	-2.20	.036	-0.42
25	-0.004	0.013	-1.76	.090	-0.33
26	-0.003	0.009	-1.66	.107	-0.31
27	-0.001	0.010	-0.44	.660	-0.08
28	-0.003	0.012	-1.22	.233	-0.23
35	0.000	0.020	-0.07	.947	-0.01
36	-0.003	0.016	-1.02	.315	-0.19
39	-0.002	0.010	-1.21	.237	-0.23
46	0.004	0.018	1.09	.285	0.21
47	0.003	0.010	1.63	.115	0.31
48	0.004	0.012	1.81	.080	0.34
49	0.001	0.014	0.29	.773	0.06

Notes. p values of significant channels ($p < \alpha$ (= .05) / Meff value) are in boldface. Meff value = 8.23. *SD* = standard deviation of spatial estimates; *t* = *t*-values; *p* = *p*-values; *d* = Cohen's *d*.

2 Supplementary Figures

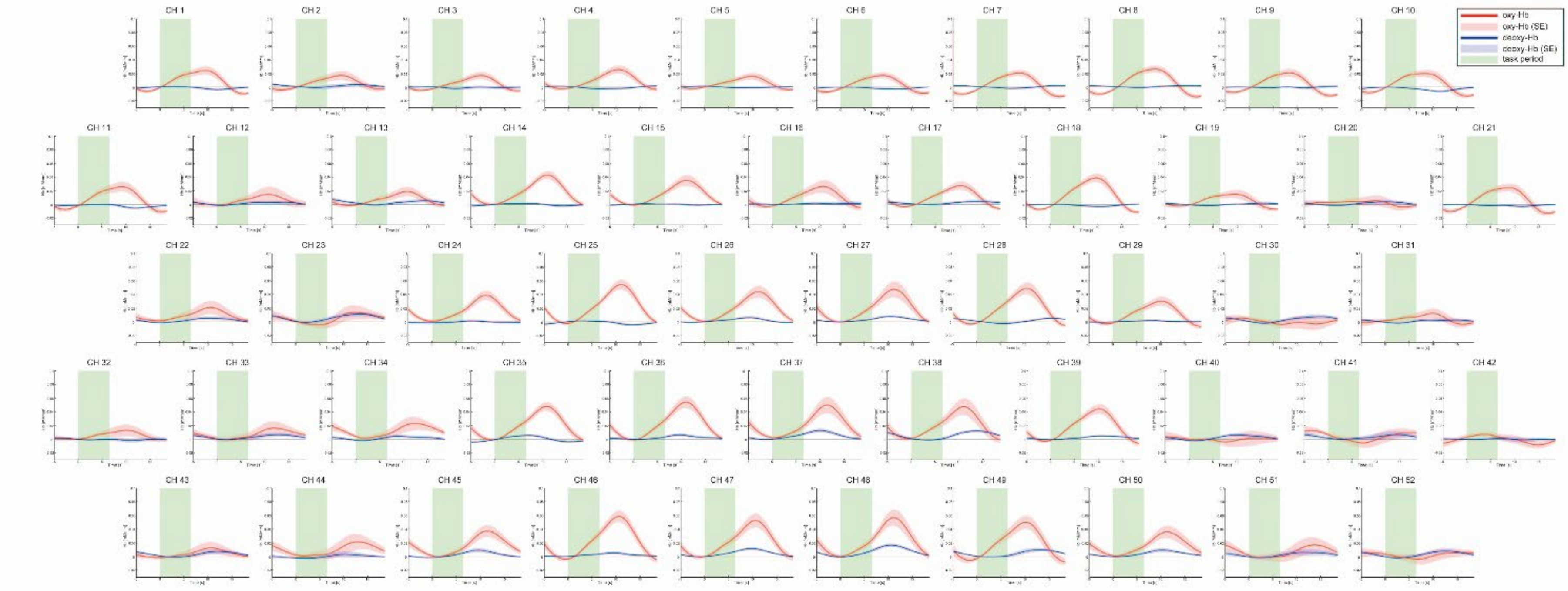

Supplementary Figure 1 Observed oxy-Hb and deoxy-Hb time-series data for all fNIRS channels during the PRTLT.

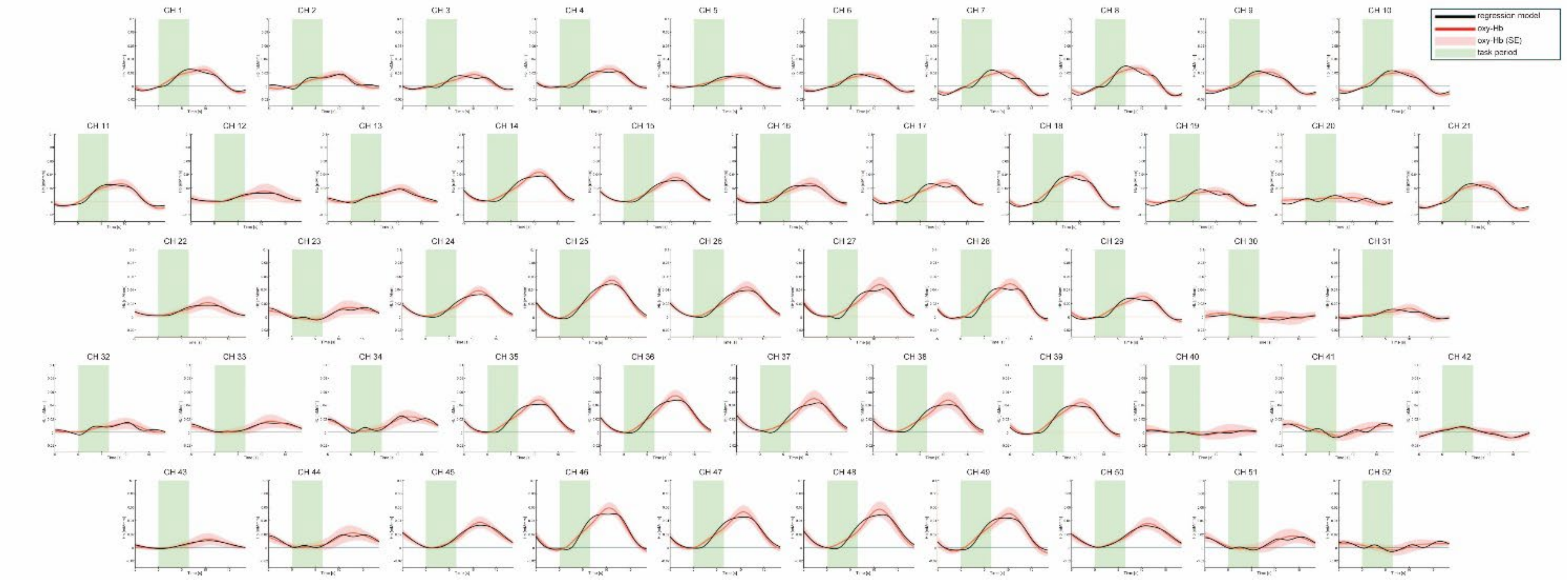

Supplementary Figure 2 Comparison between observed and predicted oxy-Hb hemodynamic time-series data for the PRTLT.

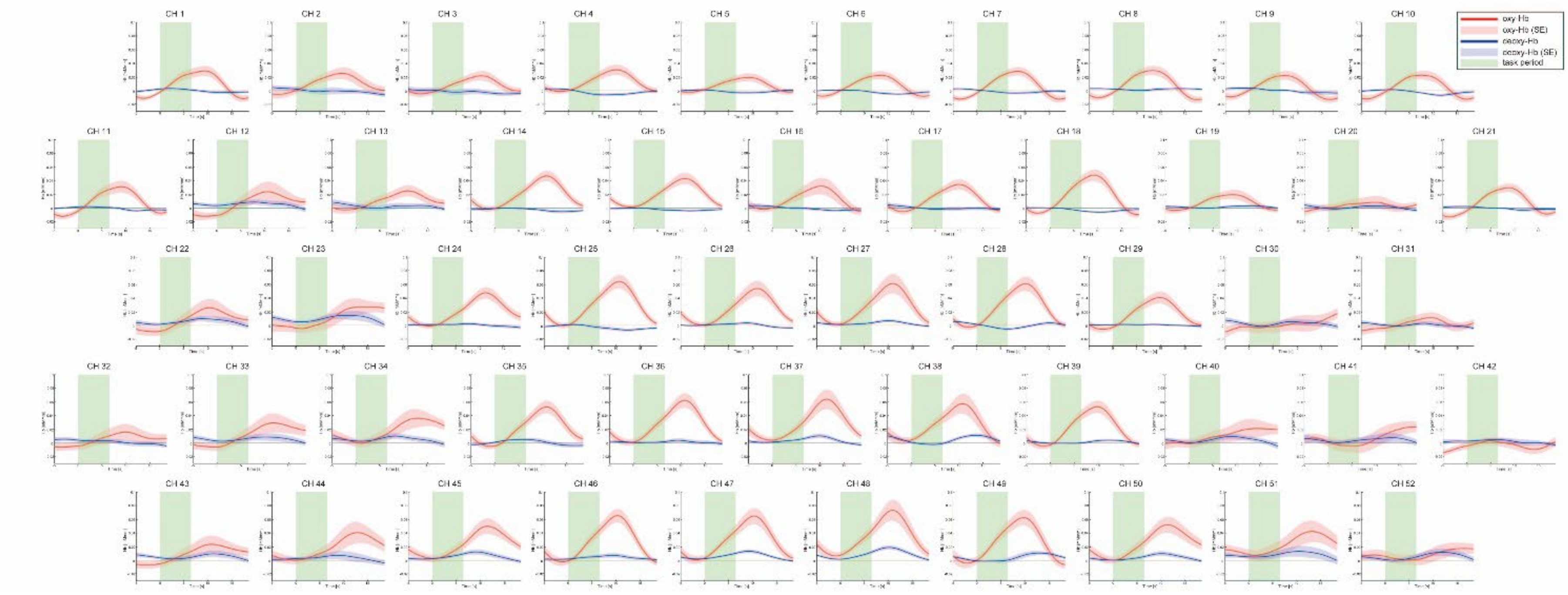

Supplementary Figure 3 Observed oxy-Hb and deoxy-Hb time-series data for all fNIRS channels in the expensive condition.

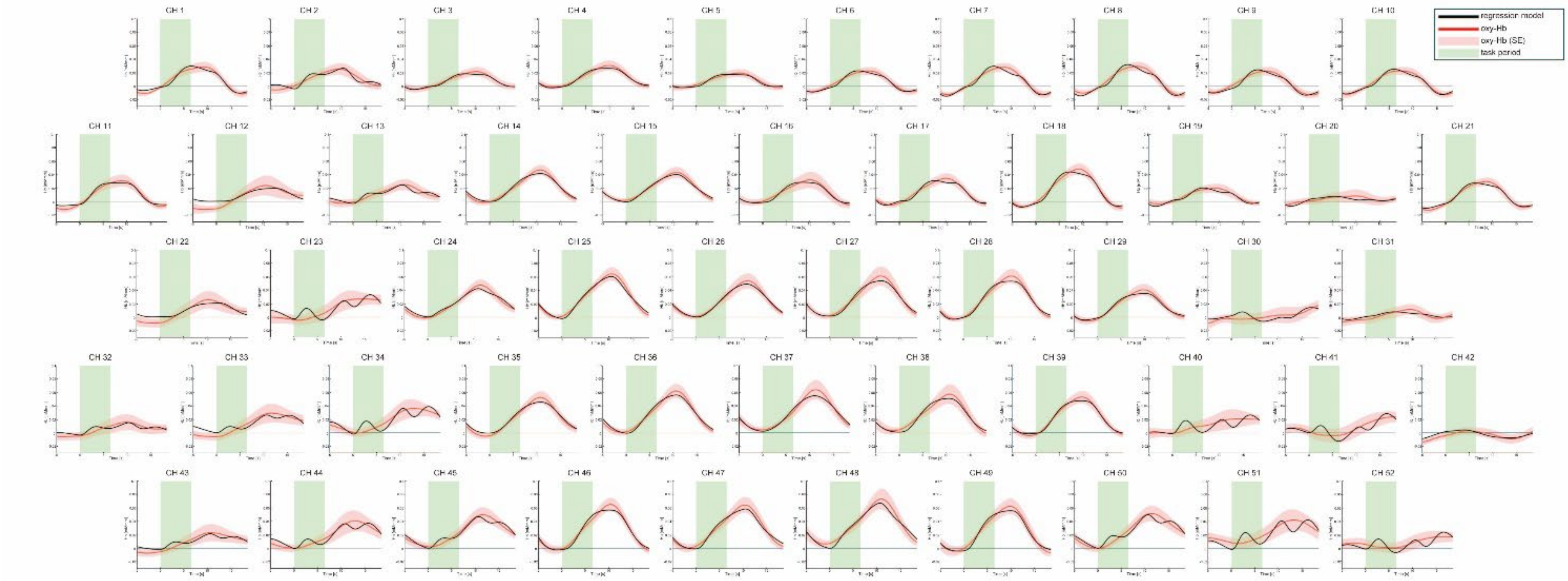

Supplementary Figure 4 Comparison between observed and predicted oxy-Hb hemodynamic time-series data in the expensive condition.

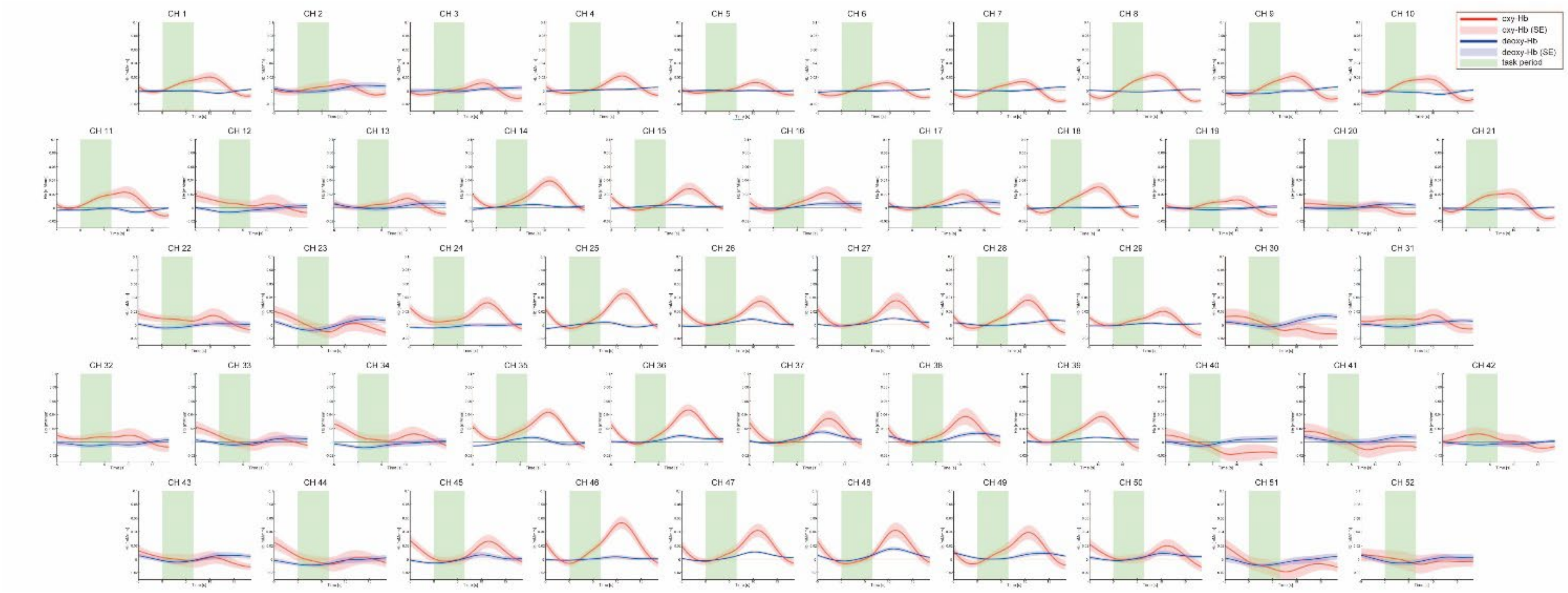

Supplementary Figure 5 Observed oxy-Hb and deoxy-Hb time-series data for all fNIRS channels in the inexpensive condition.

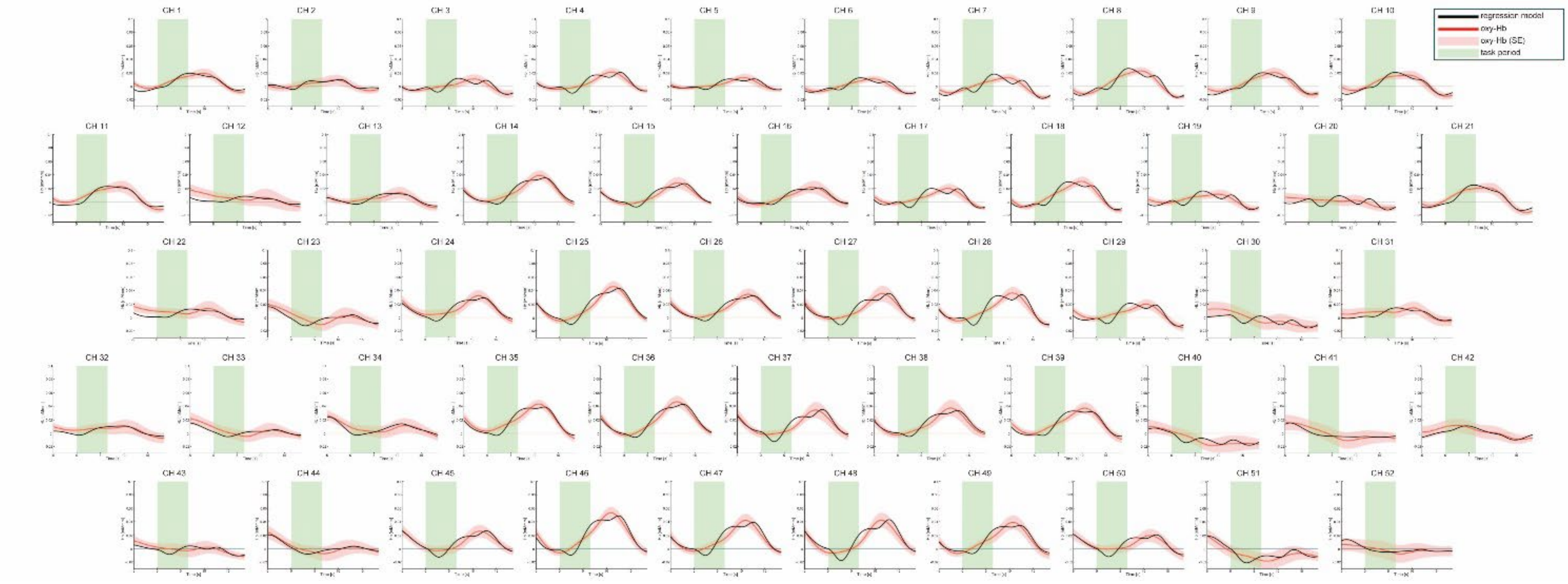

Supplementary Figure 6 Comparison between observed and predicted oxy-Hb hemodynamic time-series data in the inexpensive condition.
